# Bilingual Language Switching in the Laboratory versus in the Wild: The Spatiotemporal Dynamics of Adaptive Language Control

**DOI:** 10.1523/JNEUROSCI.0553-17.2017

**Published:** 2017-09-13

**Authors:** Esti Blanco-Elorrieta, Liina Pylkkänen

**Affiliations:** ^1^Department of Psychology and; ^2^Department of Linguistics, New York University, New York, New York 10003, and; ^3^NYUAD Institute, New York University Abu Dhabi, Abu Dhabi, United Arab Emirates

**Keywords:** adaptive cognitive control, bilingualism, language control, language switching, magnetoencephalography, prefrontal cortex

## Abstract

For a bilingual human, every utterance requires a choice about which language to use. This choice is commonly regarded as part of general executive control, engaging prefrontal and anterior cingulate cortices similarly to many types of effortful task switching. However, although language control within artificial switching paradigms has been heavily studied, the neurobiology of natural switching within socially cued situations has not been characterized. Additionally, although theoretical models address how language control mechanisms adapt to the distinct demands of different interactional contexts, these predictions have not been empirically tested. We used MEG (RRID: NIFINV:nlx_inv_090918) to investigate language switching in multiple contexts ranging from completely artificial to the comprehension of a fully natural bilingual conversation recorded “in the wild.” Our results showed less anterior cingulate and prefrontal cortex involvement for more natural switching. In production, voluntary switching did not engage the prefrontal cortex or elicit behavioral switch costs. In comprehension, while laboratory switches recruited executive control areas, fully natural switching within a conversation only engaged auditory cortices. Multivariate pattern analyses revealed that, in production, interlocutor identity was represented in a sustained fashion throughout the different stages of language planning until speech onset. In comprehension, however, a biphasic pattern was observed: interlocutor identity was first represented at the presentation of the interlocutor and then again at the presentation of the auditory word. In all, our findings underscore the importance of ecologically valid experimental paradigms and offer the first neurophysiological characterization of language control in a range of situations simulating real life to various degrees.

**SIGNIFICANCE STATEMENT** Bilingualism is an inherently social phenomenon, interactional context fully determining language choice. This research addresses the neural mechanisms underlying multilingual individuals' ability to successfully adapt to varying conversational contexts both while speaking and listening. Our results showed that interactional context critically determines language control networks' engagement: switching under external constraints heavily recruited prefrontal control regions, whereas natural, voluntary switching did not. These findings challenge conclusions derived from artificial switching paradigms, which suggested that language switching is intrinsically effortful. Further, our results predict that the so-called bilingual advantage should be limited to individuals who need to control their languages according to external cues and thus would not occur by virtue of an experience in which switching is fully free.

## Introduction

Bilingualism is a complex and multifaceted life experience occurring within a diverse social environment. Hence, a central feature of multilingual communication is bilingual individuals' ability to fluently accommodate to their interlocutor's language background. For instance, when a group of bilinguals with similar linguistic backgrounds interact, language switching is typically rampant because the conversational context poses no limitation on language choice. However, when conversing with a monolingual, a bilingual is likely to stick to the language of the monolingual interlocutor and only switch if speaking to a different person with a different language background. Consequently, the nature and degree of language switching are heavily determined by social context.

A substantial body of research on the brain bases of language switching has identified the prefrontal cortex (PFC) and anterior cingulate cortex (ACC) as primary centers of language control networks (Rodriguez-Fornells et al., 2002; Crinion et al., 2006; Blanco-Elorrieta and Pylkkänen, 2016). Importantly, the same areas have been implicated for cognitive control more generally (MacDonald et al., 2000; Braver et al., 2003; Aron et al., 2004), suggesting that language switching may in many ways be similar to nonlinguistic task switching. However, how these control networks adapt to, and operate under, different conversational conditions remains unknown. Further, the stark contrast between the apparent ease of switching in the “real world” and the effortful switching elicited by typical laboratory paradigms, commonly involving artificial color cues for switching, calls into question the extent to which the identified networks are relevant for naturally occurring language switching.

**Figure 1.**
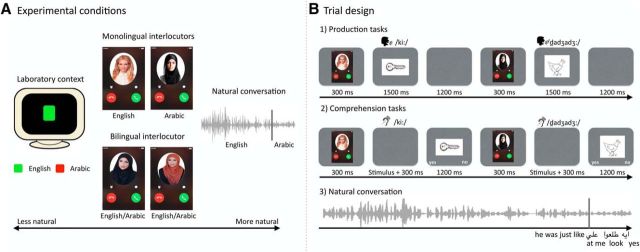
***A***, Different experimental conditions varying from less natural to more natural contexts. ***B***, Trial design for production and comprehension laboratory tasks as well as for the natural conversation. In production, participants were asked to name the drawing as quickly and as accurately as possible in the language that matched the cue they had just seen. In comprehension, participants had to judge via button press whether an auditorily presented word and a subsequently presented picture matched. In the natural conversation, participants listened to snippets of a real conversation between two Arabic-English bilinguals.

To address these questions, Arabic-English bilingual participants performed maximally parallel language switching tasks in production and comprehension while presented with a picture of either a bilingual individual (languages could be interleaved freely) or two monolingual individuals (only one language could be used with each interlocutor). To directly compare how switching in these contexts relates to artificially cued switching as used in previous literature, participants also performed the same tasks following arbitrary color cues for language that replaced the facial cues of the more natural task. Finally, to assess the overlap between these tasks and the processing of language switches contained within natural, connected bilingual speech, our participants also listened to fragments of real conversations between two bilingual individuals containing frequent language switches (Fig. 1*A*). The duration and boundaries of these clips were selected such that the dialogue in each fragment was thematically self-contained and naturally flowing. In production, participants were presented with 96 pictures standardized for psycholinguistic variables and were asked to name them as quickly and as accurately as possible in the language that matched the cue they had just seen. In comprehension, participants judged via button press whether an auditorily presented word and a subsequently presented picture matched. In the natural conversation, participants passively listened to the dialogue and were asked to answer a comprehension question about it at the end (Fig. 1*B*). Cortical activity was recorded with MEG and analyzed after stimulus presentation, before the onset of motor artifacts. Source localized neural activity was analyzed in the dorsolateral prefrontal cortex (dlPFC), ACC, and left inferior frontal gyrus (LIFG) for both production and comprehension, as well as in the auditory cortex for the comprehension tasks. We capitalized on the temporal resolution of MEG to track the millisecond-by-millisecond unfolding of neural activity during the comprehension of completely natural speech.

## Materials and Methods

### 

#### Main experiment

##### Participants.

Nineteen right-handed Arabic-English bilingual individuals participated in the experiment (8 male, 11 female, mean ± SD, 21.21 ± 3.53 years). All participants were native speakers of Arabic with a high knowledge of English (8.45 ± 1.01 in a 1–10 scale). They all came from Arab families but had always lived in an Arabic-English bilingual environment (exposure to Arabic, 41.72%; exposure to English, 58.27%, SD 15.84%) and were enrolled in an English-speaking university. Mean ages of acquisition were 1.26 years for Arabic (SD = 0.93) and 5.11 years (SD = 3.4) for English. Information about their language use and proficiency level was gathered with a modified version of the language background questionnaire of Marian et al. (2007). All subjects were neurologically intact with normal or corrected-to-normal vision and all provided informed written consent.

#### Stimuli and experimental design

The experiment consisted of three scenarios: (1) bilingual-interlocutor-context, (2) monolingual-interlocutor-context, and (3) color-cued-context, which were all performed in both production and comprehension, yielding six experimental conditions. All stimuli were taken from the only normative database for Arabic nouns (Khwaileh et al., 2014), which contains graphical depictions for 186 objects that have been standardized for psycholinguistic variables. To keep confounding linguistic variables controlled across languages, we eliminated morphologically complex words (i.e., those including suffixes, prefixes, and pluralizing morphemes) and compound words from this list. Additionally, to make sure motor demands remained constant across languages, we also rejected items that were not either monosyllabic or disyllabic in both languages, leaving 96 items in the list, which constituted our stimulus set. This stimulus set was then evaluated via an online questionnaire by 36 Arabic-English bilinguals, who were presented with the picture corresponding to each stimulus and rated on a 0% to 100% scale whether the word for that item came to mind quicker in English, in Arabic, or somewhere in between. For instance, if in their general life they would always use the English word to refer to a *plug*, they would select 100% English, 0% Arabic for this item. The purpose of this assessment was to ensure that there was an even distribution of items across both languages such that: (1) when speaking with the bilingual interlocutor, both languages would be used; and (2) we would not artificially confound the experiment by selecting a set of items that are only ever named in one of the two languages. The analysis of the ratings of the online questionnaires revealed that our stimuli were normally distributed across the languages, with 57% of our stimuli being equally likely to be named in English or Arabic, 25% being more likely to be named in English, and 17% being more likely to be named in Arabic. This distribution did not significantly vary from a normal distribution (Jarque-Bera test failed to reject the alternative hypothesis that the data do not follow a normal distribution, *p* = 0.5).

Each experimental item was presented once in each condition. Thus, each experimental condition contained 96 trials, and the experiment consisted of 576 trials in total. Experimental conditions were further divided into four blocks of 24 items to form experimental blocks. Items within blocks and blocks along the experiment were pseudorandomized following three constraints: (1) two blocks of the same task never appeared consecutively; (2) at least half of the experimental items were presented between two repetitions of the same item; and (3) each item was presented in production and comprehension in alternation. Participants were informed at the beginning of each block about the task and context of the upcoming block.

Following previous research (Blanco-Elorrieta and Pylkkanen, 2016), these items were presented in one of three types of trials: (1) trials in which the language of the target item differed from that of the preceding trial (Switch trials), (2) trials in which the language of the target was identical to that of the preceding trial but immediately followed a switch trial (Switch+1 trials), and (3) trials in which the targets' language was identical to that of the preceding trial and did not follow a switch trial (NonSwitch trials). All controlled experimental conditions contained an equal number of trials of each type (Switch, Switch+1, and NonSwitch), presented half of the times in English and half in Arabic. In the bilingual-interlocutor-context, because participants were allowed to choose the language of their choice for every item, the condition of the trial was coded after the experiment by listening to participants' responses. Consequently, the number of trials per condition (Switch, Switch+1, and NonSwitch) was not controlled in this experimental condition. To address this issue, which could result in varying signal-to-noise ratio differences across conditions and faulty estimation of the minimum norm estimates, we equalized the epoch count for all conditions before analysis.

One aspect of our design was that previous exposure to an item in one language could affect the language that was selected in the bilingual-interlocutor-context. To minimize this possible bias toward a given language, the number of items between repetitions of the same stimulus was at least half of the experimental items, which results in at least 2 whole blocks between two repetitions of the same item. Additionally, because the order of the tasks and the assignment of items to each block were randomized across participants, it was completely unsystematic whether they would have to comprehend a given item before producing it spontaneously and, also, which item(s) these would be. Hence, across participants, the effects of language priming for any given word should have diluted. However, it is also worth mentioning that in the Bilingual-context condition, we were interested in measuring brain activity while participants used the name of the object in whichever language was the easiest for them to use. Hence, whether this easier access to the word in a given language was transient (as a factor of having heard the word in a given language at some previous point in the experiment) or a stable preference for that item in that language, was not meaningful for our experimental purposes.

In the production tasks, participants named the pictures that were presented on the screen following the rules of the conversational context. In the bilingual-interlocutor-context, participants were presented with one of two pictures of a bilingual interlocutor, and they could freely choose the output language. In the Monolingual-interlocutor-context, participants were presented with a picture of one of two monolingual interlocutors following a random fashion, and participants had to choose the language that matched the interlocutor to produce their response. In the laboratory color-cued task, participants saw a green or a red square, and they had to choose the language following the color cue. Color-language associations were counterbalanced across participants (Fig. 1*A*,*B*).

In comprehension tasks, participants were presented with a cue followed by an auditory stimulus. Their task was to comprehend the stimulus and press a button to judge whether a subsequently presented picture matched the word they had just heard. As in production, the cues indicating the target language could be a picture of a bilingual interlocutor, alternating pictures of two monolingual interlocutors or color cues (Fig. 1*A*). Although the relations between color and language in the Laboratory condition were arbitrary, thus potentially recruiting rather general decision-making processes, we have previously shown that, even in this condition, the comprehension of language switches engages a different network from nonlinguistic decision processes (Blanco-Elorrieta and Pylkkänen, 2016).

All cues were matched for size and brightness, and interlocutors' pictures were presented within an iPhone calling screen to make the scenario as realistic as possible. Before the beginning of the experiment, participants were acquainted with the interlocutors by reading the personal story that led to their knowledge of English and/or Arabic. Importantly, these stories very faithfully mirrored the background stories that our participants frequently encounter in their everyday life in the United Arab Emirates, such that participants could quickly relate these interlocutors to their life experience and bring linguistic attitudes from their own lives into the experiment.

Three Arabic-English bilingual speakers recorded all auditory stimuli in a single session using a Neumeann U87 microphone and Avalon VT-737SP preamplifier. The speakers read each word three times, and the second production of the word was always selected to allow for consistent intonation across stimuli. The words recorded by each speaker were assigned to one of the three contexts following a Latin-squared design across participants to avoid voice feature differences confounding our results. Amplitude for all the recordings was equalized to 70 dB sound pressure level using Praat. The duration of each stimulus was 564 ± 163 ms.

In addition to the different laboratory tasks varying in naturalness of the context, we studied the effects of language switching in natural speech by having participants listen to clips of a real conversation. These natural conversation snippets were extracted from a recorded conversation between two native Arabic-English bilingual females. The speakers provided written consent for the conversation to be recorded but to keep the language switches as natural as possible, the speakers were not aware of the purpose of the recording until after the recording had concluded. This conversation was 3 h long, and the experimenters extracted five snippets of 1 min each where frequent code switches occurred. An Arabic-English bilingual then transcribed these snippets, and the experimenters annotated language switches contained within these recordings using Praat. In total, these recordings contained 70 language switches (35 switches from English to Arabic and 35 switches from Arabic to English). A total of 70 control trials for these natural switches were extracted from within the conversations by selecting maximally similar discourse units in which no language switch occurred. In case careful selection was not sufficient to eliminate all possible additional differences between the Switch and the NonSwitch speech fragments, potentially confounding variables such as (1) speaker change, (2) discourse boundary, and (3) language of the preceding and following speech were coded and regressed out in the statistical analyses. These recordings were also equalized to 70 dB sound pressure level using Praat.

#### Procedure

Before the MEG recording, each subject's head shape was digitized using a Polhemus dual-source handheld FastSCAN laser scanner. MEG data were collected in the Neuroscience of Language Laboratory in NYU Abu Dhabi using a whole-head 208 channel axial gradiometer system (Kanazawa Institute of Technology, Kanazawa, Japan) as subjects lay in a dimly lit, magnetically shielded room. Vocal responses were captured with an MEG-compatible microphone (Shure PG 81). In the production contexts, trials began with the presentation of the interlocutor or the color cue (300 ms), followed by the presentation of the stimulus picture. Stimuli remained onscreen until speech onset with a 1500 ms timeout and participants were then given 1000 ms to finish speech before the next trial began. Participants were instructed to respond as quickly and accurately as possible. MEG data were recorded during planning for production, before motion artifacts, and participants were allowed to blink after naming the stimulus.

In the comprehension tasks, the trial also began with the presentation of the interlocutor or the color cue (300 ms). Then, participants were presented with the auditory stimuli while looking at a blank screen. The blank screen remained onscreen for 300 ms after the end of the auditory stimulus and was followed by the presentation of a picture. Participants were then given 2000 ms to make a judgment via button press about whether the auditory and the visual stimulus matched. For all participants, the right button indicated mismatch and the left button indicated match. After the button press, a blank screen appeared for 300 ms, and then the next trial began (Fig. 1*B*). MEG data were acquired during auditory stimulus presentation to capture activity elicited by perceiving the language switch. The 300 ms lapse between the end of the auditory stimulus and the presentation of the visual stimulus was included to avoid capturing task-related activity in our epoch (i.e., decision making and mismatch detection). However, it is likely that participants processed the language switch during this lapse. Consequently, the behavioral reaction times measured from visual stimulus presentation were not informative of online switching. Therefore, only accuracy was considered a reliable measure of participants' switching performance in comprehension tasks. Participants were allowed to blink during visual stimulus presentation, before the button press that initiated the following trial.

#### Data acquisition and preprocessing

Data were preprocessed and analyzed with MNE-Python (Gramfort et al., 2013, 2014; RRID: SCR:005972) and Eelbrain package (https://pythonhosted.org/eelbrain). MEG data were recorded at 1000 Hz (200 Hz low-pass filter), noise reduced via the continuously adjusted least-squares method (Adachi et al., 2001) in MEG Laboratory software (Yokogawa Electric and Eagle Technology) and epoched from 100 ms before interlocutor cue (400 ms before stimulus) to 600 ms after critical stimulus onset. All epochs containing sensor values exceeding 2500 fT/cm at any time after noise reduction were automatically rejected. For artifact rejection, we applied an independent component analysis to our raw data, and components corresponding to blinks and heartbeats were removed. A strict artifact rejection routine used in previous MEG production studies (Pylkkänen et al., 2014; Blanco-Elorrieta and Pylkkänen, 2016) was followed to ensure that oral artifacts were not contaminating our data. Specifically, we: (1) removed all trials that contained naming latencies within our epoch; (2) rejected all individual epochs that contained amplitudes >2500 feet/cm for any sensor after noise reduction; (3) visualized all individual epochs before averaging and rejected any epoch that contained sudden increases in the magnitude of the signal caused by artifacts (be it muscular movements or else); and (4) we applied a 40 Hz low pass filter that should eliminate any remaining oral movement from our data, given that the gamma-frequency range (>40 Hz) is reportedly the one affected by muscle artifact contamination, such as phasic contractions (Yuval-Greenberg and Deouell, 2009; Gross et al., 2013). In addition, trials corresponding to behavioral errors or response times within the length of our epochs were also excluded from further analyses. To account for potential power differences across conditions with different number of rejected trials, we equalized the epoch count for all conditions before averaging. This was done by first identifying the condition of the experimental design with the smallest number of observations and then, for each condition, only keeping the observations up to that number. Together, this resulted in the exclusion of 19.5% of the trials (SD 3.65%), leaving 463.26 trials on average per subject (SD 21.10) and 25.72 trials per condition (SD 3.5).

To estimate the distributed electrical current image in the brain at each time sample, we used the Minimum Norm Approach (Hämäläinen and Ilmoniemi, 1994) via MNE (MGH/HMS/MIT Athinoula A. Martinos Center for Biomedical Imaging, Charleston, MA). The cortical surfaces were constructed using an icosahedron subdivision of 4 and mapping an average brain from FreeSurfer (CorTech and MGH/HMS/MIT Athinoula A. Martinos Center for Biomedical Imaging) to the head-shape data gathered from the headscanning process. This generated a source space of 5124 points for each reconstructed surface, leaving ∼6.2 mm of spacing within sources (cortical area per source = ∼39 mm^2^). Then, the boundary-element model method was used to calculate the forward solution. The 100 ms precue period was used to construct the noise covariance matrix and to apply as a baseline correction. The inverse solution for each subject was then computed from the noise-covariance matrix, the forward solution, and the source covariance matrix, and was applied to (1) the evoked response (in categorical analysis) or (2) the individual epochs (in regression analyses). The application of the inverse solution determined the most likely distribution of neural activity in the source space. Minimum norm current estimates were computed for three orthogonal dipoles, of which the root mean square was retained as a measure of activation at that source (thus, the orientation of the dipole was free unsigned). The resulting minimum norm estimates of neural activity were transformed into normalized estimates of noise at each spatial location using the default regularization factor (SNR = 3). Hence, we obtained noise-normalized statistical parametric maps (SPMs), which provide information about the statistical reliability of the estimated signal at each location in the map with millisecond accuracy. Then, those SPMs were converted to dynamic maps (dSPMs). To quantify the spatial resolution of these maps, the point spread function for different locations on the cortical surface was computed. The point spread is defined as the minimum norm estimate resulting from the signals coming from a current dipole located at a certain point on the cortex. The calculation of the point spread function following the approach of Dale et al. (2000) reduces the location bias of the estimates, in particular, the tendency of the minimum norm estimates to prefer superficial currents (i.e., their tendency to misattribute focal, deep activations to extended, superficial patterns). Hence, by transforming our minimum norm estimates to dSPM, we obtained an accurate spatial blurring of the true activity patterns in the spatiotemporal maps (Dale et al., 2000).

#### Analyses

##### Behavioral data.

In the comprehension tasks, incorrect button presses were coded as errors for accuracy measures. Reaction times were not analyzed because they were elicited after a substantial delay from the stimulus onset and were consequently not informative of online switching (see Procedure). In the production tasks, participants' vocal responses were evaluated for each trial and reaction times corresponding to erroneous responses [incorrect naming, verbal disfluencies (i.e., utterance repairs, stuttering), and nonresponses] were excluded from further analysis. In addition, trials following participants' errors were also excluded if such errors altered the type of subsequent trials (e.g., in a “switch, nonswitch” sequence where item 1 was labeled “Switch to Arabic” and item 2 “NonSwitch in Arabic,” item 2 was excluded if the participant erred in item 1 by naming it in English, given that this would imply that even if item 2 was correctly named in Arabic, it would not be “NonSwitch” anymore). Naming latencies < or >2.5 SD from the mean were also discarded. Reaction times for production and accuracy rates for production and comprehension were averaged over subjects and over trials per condition and subjected to 3 × 3 ANOVAs with the main factors Context (Bilingual, Monolinguals, or Laboratory) and Switch (Switch, Switch+1, NonSwitch). If an interaction was found, pairwise comparisons were also examined with paired *t* tests (two-tailed), applying Bonferroni procedure to correct for multiple comparisons. Behavioral switch costs were determined by the differences in naming latencies between switch and nonswitch trials within each of the tasks (RT switch_bilingual production_ − RT nonswitch_bilingual production_).

#### Region of interest (ROI) analyses

Planned ROIs were defined following previous studies on language switching that have found the dlPFC and the ACC to be involved in language switching (Hernandez et al., 2000, 2001; Rodriguez-Fornells et al., 2005; Crinion et al., 2006; Wang et al., 2007; Abutalebi et al., 2008). Additionally, because the dlPFC neighbored Broca's area on the left hemisphere, we also included this region in the analysis. The dlPFC included Brodmann areas (BAs) 9, 10, and 46, the ACC contained BA24, BA32, and BA33 and Broca's area included BA44, BA45, and BA47. The vertices within each of these labels were as defined in PALS_B12_Brodmann parcellation (https://surfer.nmr.mgh.harvard.edu/fswiki/PALS_B12). Although some subcortical structures, such as the basal ganglia, have been hypothesized to similarly mediate language switching, the use of MEG prevented us from analyzing activity in these areas. All these ROIs were included as a single mask in separate analyses for production and comprehension. Additionally, given previous hypotheses that the auditory cortex may initiate the perceptual identification of the phonological and/or prosodic features that identify auditory input as belonging to a different language (Blanco-Elorrieta and Pylkkänen, 2016), we ran an analysis for the comprehension data also in the auditory cortex, creating a mask that contained the transverse temporal, superior temporal and supramarginal gyri. The vertices for each of these labels were defined as in the Desikan–Killiany Atlas (https://surfer.nmr.mgh.harvard.edu/fswiki/CorticalParcellation). To control for potential cue differences in the visual cortex, we ran a *post hoc* analysis, which included BAs 17, 18, and 19 bilaterally (vertices available at https://surfer.nmr.mgh.harvard.edu/fswiki/PALS_B12). Last, to test for effects that fell outside our primary ROIs, we ran an analysis including the whole left hemisphere.

In each analysis, we analyzed current estimates using nonparametric spatiotemporal cluster tests. For each statistical test, a map of *F* or *t* values was computed over sources and milliseconds. These maps were thresholded at a value equivalent to *p* = 0.05 (uncorrected); then, clusters were computed from adjacent values in space and time that surpassed our cutoff threshold. If a cluster consisted of a minimum of 10 vertices and lasted for at least 25 ms, the *t* values within this cluster were summed, resulting in a cluster-level statistic. We then permuted the data 10,000 times, and each permutation involved shuffling condition labels at random and recomputing the cluster statistic of the permuted data to form a distribution of cluster-level *t* or *F* values of the maximum cluster-level statistic for each permutation (Maris and Oostenveld, 2007). The permutation tests were conducted in the window between the presentation of the cue and stimulus (−400 to 0 ms) and after stimulus presentation (100–400 ms), including all analyzed areas. Pairwise differences within the cluster were computed using paired-samples *t* tests and corrected with false discovery rate over tests.

#### Single-trial regression analysis

To maximize statistical power and account for possible confounding factors in our analysis of the natural conversation, we coded conditions as categorical variables within a regression analysis. Running a single trial analysis in combination with a spatiotemporal permutation cluster test involves three stages (for a schematic depiction, see Gwilliams et al., 2016, their Fig. 2). First, we ran an ordinary least-squares regression using the source estimates of each trial within a selected region as the dependent measure. The data were in the form of 3D matrices with the shape: space (number of vertices in the tested ROI) × time (number of milliseconds within the window interest) × item (number of trials). The regression model included variables of interest (specifically, language switch, speaker change, preceding language, following language and discourse boundary), a random intercept, and a nuisance variable (order of conversation snippets). The variable speaker change indexed whether the language switch occurred when a different speaker spoke, and discourse boundary tracked the level of the linguistic unit at which a given language switch occurred (quotation, sentence, clause, phrase, word; for examples, see Fig. 6*C*). This model was run on each subject's data separately, resulting in a β coefficient for each source, millisecond, and variable of interest for each subject. Then, we ran a one-sample *t* test on the distribution of β values across subjects for each variable at each source and time point, to test whether their value was significantly different from zero. This resulted in a matrix of *t* values, with a dimension for each source and time point. This matrix was then subjected to a cluster-permutation test (10,000 permutations) as described in ROI analyses, and clusters with a final *p* value <0.05 after correction were considered to reflect a reliable impact of that predictor on brain data.

#### Multivariate pattern analysis (MVPA)

Our MVPA was adapted from King et al., (2014) and was implemented to track the temporal dynamics of our participants' representation of the various interlocutor types. This analysis was based on the assumption that specific context types should engage specific configurations of cortical currents, which then could be tracked in the sensor topographies of the measured magnetic fields. The goal of this MVPA was to construct, at each time point and for each subject separately, a classifier that specifically isolated such a topography for the three interactional contexts we investigated: bilingual-interlocutor-context, monolingual-interlocutors-context, and color-cued-context. In other words, we aimed to extract the pattern of MEG activity that distinguished the different interlocutor types. Contrary to the univariate analyses, this analysis was run in sensor space because, when constraining the source reconstruction to a set of ROIs, or to an a priori defined source model, brain activity that is not coming from the modeled areas may be erroneously projected onto these sources (Gross et al., 2013). Hence, running multivariate analysis in source space for MEG is not recommended as one could end up including in the classification procedure data that are highly correlated across sources or retrieving signals that are outside the area of interest.

For each within-subject analysis, a fivefold cross-validation procedure was implemented. Within the cross-validation, MEG signals were normalized for each classifier separately. Stratified cross-validation balanced the proportion of each class (bilingual-interlocutor-context, monolingual-interlocutors-context, and color-cued-context) in each fold. A linear support vector machine for each fold and at each time point was then fitted on four-fifths of the trials (i.e., the training set). Each support vector machine aimed at finding the hyperplane (i.e., the topography) that best discriminated trial type at each time sample. This analysis captured evoked activity phase-locked to the presentation of the stimulus. Following original analyses by King et al., (2014), the regularization parameter (C) was fixed to 1. We then computed classification accuracy by testing an independent test set (1/5) and the support vector machine outputted a categorical output (i.e., discrete prediction: Bilingual, Monolinguals, or Color-cue context). Last, to equalize the contribution of each of these categories in the definition of the hyperplane (i.e., topography), a sample weighting procedure was applied in proportion to the classes (Bilingual, Monolinguals, or Laboratory context). All multivariate analyses were performed with MNE's Scikit-Learn toolbox. We did not reduce the dimensionality of our data because we only had the 208 features corresponding to each channel.

#### Generalization across time

Each classifier's performance was evaluated both on its accuracy at the time point at which it had been trained and on its ability to generalize across other time samples. Hence, after a classifier had been fitted to each time point of the trial (*t*), each classifier was tested on its ability to discriminate different contexts at any time *t′*. This method led to a [training time × testing time] temporal generalization matrix (see Fig. 7*A*,*B*, left). Decoding accuracy was estimated using an accuracy score. Given that our analysis was a multiclass classification, this accuracy estimate was equal to the Jaccard similarity coefficient score for pairs of label sets. Classifiers trained and tested at the same time point correspond to the diagonal of this *t2* matrix and are termed diagonal decoding. Classifier performance on time points different from the time points at which it was trained is referred to as off-diagonal decoding. The cross-validation and the temporal generalization analyses were independent: the trials used in the training set at time *t* were never included in the generalization at time *t′* as consecutive time samples.

#### Statistics on MVPA classification accuracy

Statistical tests conducted to assess the reliability of our classification accuracy were conducted as follows. Mean and SD of the classifier at each time point were estimated over classifier accuracy over participant distribution. Then, we contrasted mean accuracy of the classifier at each time point against classification chance level (in this case, Chance accuracy = 0.33) using a one-sample *t* test. Correction for multiple comparisons over time was applied as specified by Benjamini and Hochberg (2001). We report as reliably classified activity the first time point in a sequence of at least 10 consecutive time points for which classification accuracy significantly differed from chance level at an α of *p* < 0.05 after correction for multiple comparisons. CIs (95%) for the sample classification mean accuracy were constructed over subject accuracy distribution. The topography for the millisecond with the highest classification accuracy was then source localized to establish where in the cortex the accurately classified signals were emerging from.

#### Proficiency regression

To investigate whether language background factors that had previously been identified to influence language switch costs were having a significant effect on the magnitude of our switch effect, we ran a multiple linear regression on the interaction cluster identified in the dlPFC and ACC. Specifically, we assessed whether differences in the age of acquisition, language proficiency, or language exposure to English and Arabic could predict the switch effects we observed during the spatiotemporal cluster analysis in production. Information about these variables was collected with the language background questionnaire described in Participants and the predictor indexes were calculated as follows:

1. Difference in age of acquisition: absolute value of the difference between the age of acquisition of English and Arabic.
2. Difference in proficiency: we calculated the overall proficiency of participants in each language by averaging their self-assessed proficiency in reading, writing, listening, and speaking in each language. The predictor then encompassed the absolute value of the difference in proficiency between the two languages.
3. Difference in use: participants self-assessed the percentage of time they used each language when reading, writing, listening, and speaking in each language. The overall proficiency was calculated as the average over these fours skills, and the predictor encompassed the absolute value of the difference in proficiency between the two languages.

The dependent variable indexing the overall switch cost over conditions was calculated as follows:

1. For each participant and experimental condition within production, we extracted and averaged activity over the sources and time points identified in the dlPFC and ACC interaction cluster revealed by the spatiotemporal ANOVA.
2. We calculated the switch cost for each of the contexts by subtracting NonSwitch from Switch activity.
3. Last, we summed the switch effect values for the three contexts for each participant. This resulted in a single data point that indexed the overall switch effects over the three interactional contexts for each participant.

The multiple linear regression analysis was then conducted by using Difference in age of acquisition, Proficiency, and Language use to assess whether they were reliable predictors of switch costs in the three contexts. We ensured that these predictors were not collinear with each other using Belsley's collinearity test as implemented in MATLAB (highest index = 3.25 for a default tolerance of 30; RRID: SCR_001622). The same analysis was also conducted in each of the contexts independently.

#### Brain data as predictor of reaction times

We additionally investigated whether behavioral naming latencies could be predicted based on the prefrontal and anterior cingulate activity identified as locus of the neural language switching effect. For this analysis, we first extracted and averaged the mean amplitude over the sources and milliseconds in the cluster for the neural switch effect identified in the spatiotemporal clustering ANOVA. Subsequently, we fitted a mixed-effects linear model with reaction time as the dependent variable, fixed effect of mean amplitude, and random effects of subject and item, to address whether this activity could predict behavioral switch costs. Our predictor variable was the mean dSPM activity over the switch effect cluster, and the predicted variable was behavioral reaction times for each trial.

## Results

Our analyses searched for spatiotemporal clusters reliably affected by the stimulus manipulation in a single mask containing three broad regions, all of which have previously been implicated as relevant for cognitive control and lexical retrieval: the PFC, ACC, and LIFG (Hernandez et al., 2001; Braver et al., 2003; Rodriguez-Fornells et al., 2005; Wang et al., 2007; Hikosaka and Isoda, 2010; Guo et al., 2011; De Baene et al., 2015; Weissberger et al., 2015). Production and comprehension data were analyzed separately, and further, within comprehension, the three conditions that followed a typical laboratory trial structure (involving the bilingual, monolingual, and color cues to language in both comprehension and production, henceforth “laboratory experiment”) were first analyzed separately from the “natural speech” condition, given the rather different overall signal properties elicited by a continuous conversation compared with single words. When the cue determined the upcoming language, as in the monolingual and color contexts, language switching could in principle start already at the cue, before the lexical item was revealed. To look for such immediate shifts in attention between the two languages, we analyzed not only the activity after picture or word onset, but also the activity between cue onset and picture/word onset.

### At the cue: effects of context but not language switching in production and comprehension

**Figure 2.**
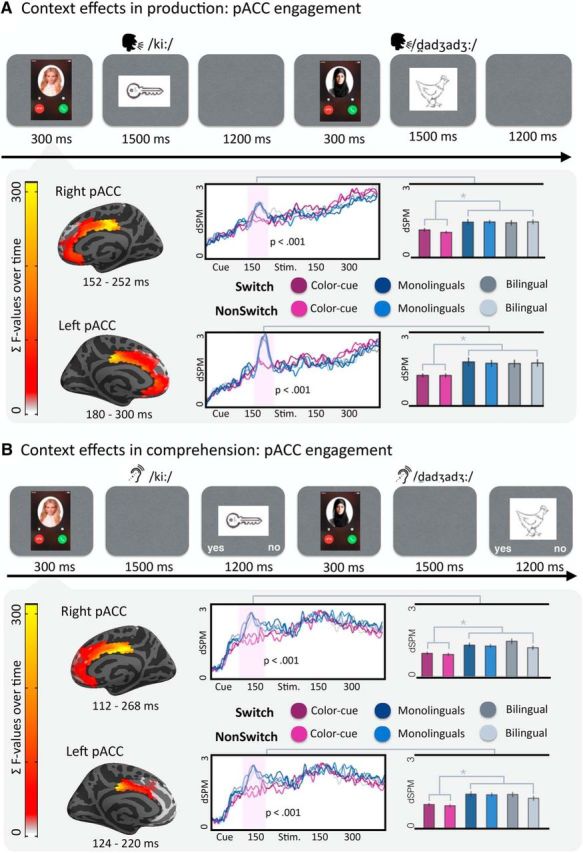
Context effects in production (***A***) and comprehension (***B***). The analysis of the MEG activity time-locked to the presentation of the cue revealed reliable clusters in the posterior part of the ACC bilaterally. In both sections, the freeSurfer average brains represent the spatial distribution of the reliable cluster (every source that was part of the cluster at some point in time is color-coded with the *F* statistic summed over the duration of the cluster). On the waveform plots, we show the time course of activity for the sources in the cluster. Shaded regions represent that the difference in activity between the tested conditions was significant at *p* = 0.05 (corrected). Significance was determined using a nonparametric permutation test (Maris and Oostenveld, 2007) performed from 0 to 300 ms after the presentation of the cue (10,000 permutations). Right, Bar graph represents the average activity per condition for the sources and time points that constitute the cluster. Pairwise significance is indicated.

The analysis at cue presentation revealed that, even in the contexts where the cue determined language choice (color-cue and monolingual contexts), cue presentation did not elicit reliable effects of switching. However, the posterior portion of the ACC showed sensitivity to the basic contrast of face versus color cue before the presentation of the auditory word or picture-to-be-named. This showed that in both production and comprehension, facial cues elicited increased activity compared with color cues in the posterior part of the ACC bilaterally (Fig. 2). This effect started slightly earlier in comprehension (112–268 ms, *p* < 0.0001, right hemisphere; 124–220 ms, *p* < 0.0001, left hemisphere) than in production (152–252 ms, *p* < 0.0001, right hemisphere; and 180–300 ms, *p* < 0.0001, left hemisphere), and did not qualitatively match the pattern of activity observed in the visual cortex, suggesting that the interlocutor effects in posterior ACC may reflect higher level representations of the interlocutors as opposed to low-level visual features of the cues themselves. In sum, although different cue types elicited distinct activity patterns, we found no evidence for language switching effects in this early time-window, before the presentation of the linguistic stimulus.

### Production: switch effects in dlPFC and ACC decrease with naturalness and spontaneity of the language switch

**Figure 3.**
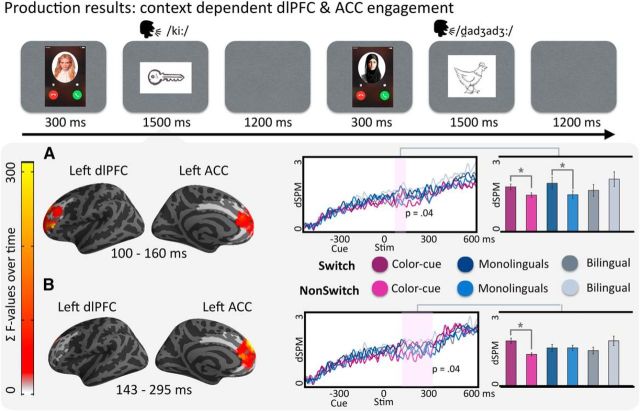
Production trial design and results. The analysis of the MEG activity time-locked to the stimulus picture revealed two temporally distinct significant interaction clusters in the left ACC and dlPFC. The freeSurfer average brains on the left-hand side represent the spatial distribution of the reliable cluster (every source that was part of the cluster at some point in time is color-coded with the sum *F* or *t* statistic). On the waveform plots, we show the time course of activity for the sources in the cluster, where 0 is the presentation of the stimulus. Shaded regions represent that the difference in activity between the tested conditions was significant at *p* = 0.05 (corrected). Significance was determined using a nonparametric permutation test (Maris and Oostenveld, 2007) performed from 100 to 300 ms (10,000 permutations). Right, Bar graph represents the average activity per condition for the sources and time points that constitute the cluster. *Pairwise significance.

Given that no switch effects were identified at the presentation of the cue, we proceeded to analyze potential switch effects emerging after the presentation of the critical stimulus to be named. In the production part of the laboratory experiment, analysis of the effects of Context (Bilingual, Monolingual, or Color-cued) and Switching (Switch or Nonswitch) revealed two reliable clusters. The first cluster was contained within the dlPFC and ACC and was modulated by whether the language switch was spontaneous or imposed by external cues. Specifically, we found that, at an early time window (100–160 ms), there was an interaction between Context and Switch that showed increased switch activity in both Color-cued and Monolingual contexts (where participants followed external cues for switching) but no increase for switching in the Bilingual context, where participants were free to choose the response language (*p* = 0.04; Fig. 3*A*). Additionally, we found a second Context × Switch interaction cluster occurring at a slightly later time window (143–295 ms, *p* = 0.04) and spatially overlapping in the ACC. This interaction was caused by increases for Switch over NonSwitch trials in the Color-cued context that were not present in either the Monolingual or the Bilingual contexts. The longer switch effect in the Color-cued context thus suggests that, even though both the Monolingual and Laboratory contexts followed external cues for switching, switching in response to a completely artificial cue requires more intense engagement of language control networks (Fig. 3*B*).

**Figure 4.**
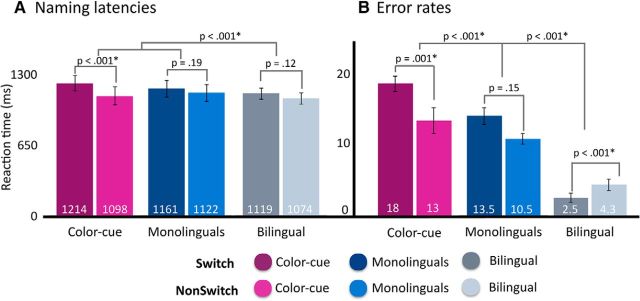
Mean reaction times (***A***) and error rates (***B***) as a function of the conversational context and performed switching condition within production tasks. The number at the bottom of each bar indicates the average value for that condition. Error bars indicate SEM.

The behavioral naming latencies matched the pattern in the neural data (Fig. 4*A*): naming was slowest in the Color-cued context (1159 ms), followed by the Monolingual (1140 ms), and finally the Bilingual (i.e., free switching) context (1094 ms; main effect of Context, *F*_(2,36)_ = 12.9, *p* < 0.0001). Specifically, the Bilingual condition was significantly faster than both the Monolingual (*t*_(18)_ = 4.15, *p* < 0.001) and the Color-cued context (*t*_(18)_ = 4.93, *p* < 0.001). Naming in the Monolingual condition was also marginally quicker than in the Color-cued context (*t*_(18)_ = 2.04, *p* = 0.052). Importantly, Context interacted with Switch (*F*_(4,72)_ = 4.84, *p* = 0.001) such that a reliable switch cost was only elicited in the Color-cued context (*t*_(18)_ = 5, *p* < 0.0001), with the Monolingual and Bilingual contexts simply trending similarly (Monolingual *t*_(18)_ = −1.34, *p* = 0.195; Bilingual *t*_(18)_ = −1.83, *p* = 0.125) (Fig. 4*A*). This result matches previous findings by Gollan and Ferreira (2009), who found balanced bilinguals to not exhibit switch costs if they were given the opportunity to choose any language to name pictures (Gollan and Ferreira, 2009, their Experiment 1, their Fig. 2). Accuracy data conformed to this pattern: participants made the most errors in the Color-cued context, followed by the Monolingual and finally the Bilingual context (*F*_(2,36)_ = 55.17, *p* < 0.0001; Fig. 4*B*). Also, Context and Switch interacted in accuracy data as well such that a reliable switch cost was found for the Color context (*t*_(18)_ = −3.64, *p* = 0.001), but not for the Monolingual (*t*_(18)_ = −1.46, *p* = 0.15) or Bilingual contexts (*t*_(18)_ = 4.37, *p* < 0.0001; Fig. 4*B*). It is worth mentioning that, even though we allowed participants to voluntarily decide when to switch in the Bilingual condition, our stimulus selection was successful in eliciting a balanced number of Switch and NonSwitch trials. In the Bilingual condition, the trials that entered the analysis across all participants were 51.9% NonSwitch (751 trials) and 48.1% Switch (695 trials). After eliminating incorrect trials, the trials entering the analysis in the Monolingual condition were 51.8% NonSwitch (545 trials) and 48.1% Switch (506 trials) and in the Color-cue condition it was 51.45% NonSwitch (529 trials) and 48.54% Switch (499 Switch trials). Hence, we ensured that the lack of a switch cost in the Bilingual condition does not stem from a dissimilar number of Switch and NonSwitch trials.

Thus, both the MEG and the behavioral data suggest that language switching is harder when the language cue is artificial and gets easier as the paradigm becomes more natural. To further examine the relationship between the neural and behavioral results, we conducted a regression analysis to assess whether we could predict behavioral reaction times for each trial based on the average activity in the sources and time points involved in the Context × Switch interaction clusters (for model details, see Materials and Methods). However, we did not find a reliable relation between the two. Last, given previous evidence showing that age of acquisition, differences in language use and differences in language proficiency can influence bilingual performance and cortical organization (Perani et al., 1998; Abutalebi et al., 2013), we performed an additional regression analysis to test whether these factors could predict the magnitude of the neural switch effect for each participant. This analysis did not reveal any such effect (Age of Acquisition: *t*_(18)_ = −1.55, *p* = 0.14; Exposure: *t*_(18)_ = −1.77, *p* = 0.1; Proficiency: *t*_(18)_ = −0.37, *p* = 0.71), possibly due to the small range of differences in these variables across our participants (for regression model details, see Materials and Methods).

### Comprehension: switch effects in dlPFC and ACC in laboratory tasks but in auditory cortex during natural speech

**Figure 5.**
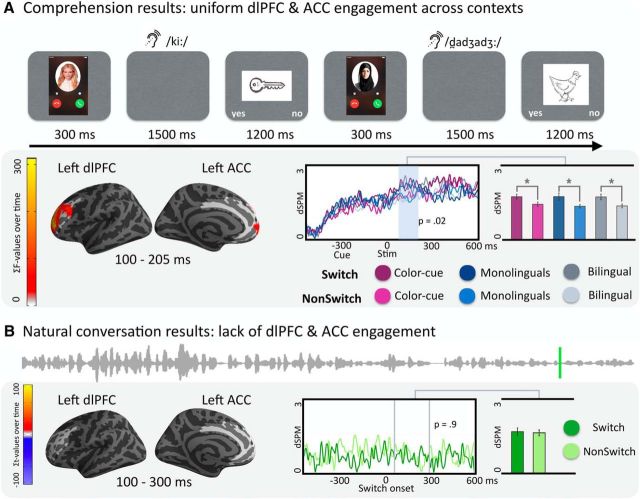
Comprehension trial design and results: ***A***, Laboratory contexts. ***B***, Natural conversation. The analysis of the MEG activity time-locked to the beginning of the auditory word revealed a significant cluster of activity in the left ACC and dlPFC, reflecting a main effect of Switch. Left, The freeSurfer average brains represent the spatial distribution of the reliable cluster (every source that was part of the cluster at some point in time is color-coded with the sum *F* or *t* statistic). On the waveform plots, we show the time course of activity for the sources in the cluster, where 0 is the beginning of the auditory stimulus. ***A***, Shaded region represents that the difference in activity between the tested conditions was significant at *p* = 0.05 (corrected). ***B***, Vertical lines indicate the analyzed window. Significance was determined using a nonparametric permutation test (Maris and Oostenveld, 2007) performed from 100 to 300 ms (10,000 permutations). Right, Bar graphs represent the average activity per condition for the sources and time points that constitute the cluster. *Pairwise significance.

While in production, the analysis of the laboratory tasks revealed that switch effects varied as a factor of the context, switch effects in comprehension were homogeneous across the three laboratory contexts (main effect of Switch: 100–250 ms, *p* = 0.02; Fig. 5*A*). These effects were observed in the left dlPFC and the anterior part of the ACC. Because previous research has suggested that bilinguals use identity of the interlocutor as a cue to predict the incoming language (Martin et al., 2016), our expectation was that increased uncertainty about the upcoming language in the Bilingual context would lead to larger switching-related activity. However, we did not identify any interaction clusters that would support this (all *p* values >0.6). No switching effects were localized in the auditory cortex either (all *p* values >0.3).

Once we had characterized the neural sensitivity to language switching in tasks that follow a typical laboratory trial structure, we investigated whether those areas were also involved in the comprehension of language switches in our natural conversation. The analysis of the natural conversation was conducted independently because the difference in signal amplitude between the auditory M100 in single-word presentation, and the amplitude of the MEG response to continuous speech was likely to lead to spurious task effects. Additionally, a targeted analysis in the time window and sources implicated in the switch effects for the three conversational contexts should maximize our chances of obtaining a similar switch effect in natural conversation, should there be one. However, this analysis revealed no parallelisms: in the natural conversation, there were no reliable activity increases for Switch fragments over Nonswitch fragments in either the dlPFC or the ACC (all *p* values >0.9; Fig. 5*B*). To statistically validate the differences between the comprehension of spontaneous speech and the comprehension of switching in a typical laboratory paradigm, we additionally ran a single analysis, including all comprehension data in the dlPFC and ACC. This analysis revealed a main effect of Task (100–400 ms, *p* < 0.001) and, as expected, a reliable interaction between Context and Switching (185–242 ms, *p* = 0.03). This interaction was led by increased engagement of the dlPFC and ACC during the comprehension of language switches in the Color-cued context (*t*_(18)_ = −3.50, *p* = 0.003), in the Bilingual context (*t*_(18)_ = −2.92, *p* = 0.009), and in the Monolingual context (*t*_(18)_ = −3.82, *p* = 0.001), but not in the Conversation context (*t*_(18)_ = −0.42, *p* = 0.67).

**Figure 6.**
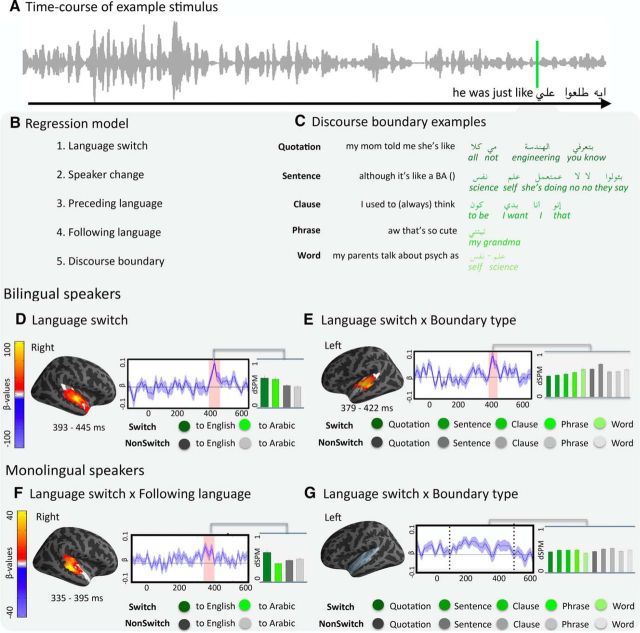
Regression analysis on the MEG data acquired during the presentation of the Natural conversation. ***A***, Waveform amplitude for an example stimulus. ***B***, Regressors in the model. ***C***, Examples of the fifth predictor, discourse boundary. ***D***, ***E***, Clusters of activity in the left and right auditory cortices, respectively, for the bilingual speakers. ***F***, ***G***, Same analyses for the monolingual speakers. The freeSurfer average brains illustrate the spatial distribution of the reliable clusters (every source that was part of the cluster at some point in time is color-coded with the sum β values). ***G***, Colored area represents the extent of the analyzed area. On the waveform plots, we show the time course of activity for the sources in the cluster, as indicated by the regression coefficients at each time point, where 0 is the beginning of the first word of the language switch. Shaded regions represent that the regression equation was significant at *p* = 0.05 (corrected). Significance was determined using a nonparametric permutation test (Maris and Oostenveld, 2007) performed from 100 to 500 ms (10,000 permutations). Right, Bar graphs represent the average activity per condition for the sources and time points that constitute the cluster. The analysis of the MEG activity was time-locked to the beginning of the first word of the code switch.

Instead of prefrontal engagement, the natural conversation elicited a reliable switch-related increase in the right auditory cortex (390–466 ms, *p* = 0.04), supporting the hypothesis in Blanco-Elorrieta and Pylkkänen (2016) that the auditory cortices should be sensitive to language switching in comprehension. A *post hoc* regression analysis was then conducted to characterize more precisely the profile of this auditory activity, aiming to test whether the increased activity observed for switching was indeed related to language switching as opposed to various nuisance variables (for regression description, see Materials and Methods). The regression model assessed the following: (1) whether the language of the preceding or subsequent speech influenced the switch effect; (2) whether having a change of speaker in addition to a language change modulated the activity; and (3) whether the nature of the discourse boundary at which the switch occurred influenced the activity cluster (i.e., whether the switch occurred at the level of a single word, phrase, clause, sentence, or third-person speech quotation; Fig. 6*B*,*C*). The analysis replicated the switch cluster in the right auditory cortex that the categorical *t* test had shown (Fig. 6*D*) and further clarified that this effect was not sensitive to the directionality of the switch. In other words, right auditory cortex activity was enhanced whether the switch was from English to Arabic or vice versa.

Importantly, the regression analysis also revealed a switch effect in the left auditory cortex. However, the left lateral switch effect interacted with Discourse Boundary (379–421 ms, *p* = 0.04), which is likely the reason why it was not revealed in the categorical analysis. The nature of this interaction was that the magnitude of the switch effect was smaller when participants switched languages at bigger discourse boundaries (e.g., at the sentential level, when speakers switched languages to produce a whole new sentence) than when participants switched languages at smaller discourse units (e.g., when a whole sentence was uttered in one language and only one single word was produced in the other language) (Fig. 6*E*). Because switches may be more likely at bigger boundaries, the left auditory cortex may thus be tracking the predictability of the switch.

One remaining possibility is that the observed switch effects could simply be due to the two languages constituting two different types of physical signals, given their distinct prosodic and phonemic properties. To test whether such low-level factors may have driven the effect, we conducted a control experiment with monolingual English speakers (*N* = 21; 9 male, 12 female; 27.45 ± 9.69 years of age), who listened to the same dialogue as the bilingual speakers. If the switch effects observed for bilinguals were indeed driven by the physical differences of the two languages, then the results obtained from the monolinguals should parallel those of the bilinguals. However, although the monolinguals also showed increased right auditory cortex activity when Arabic speech switched to English, this was not observed when English switched to Arabic, in contrast to the bilingual speakers. Thus, while in the bilinguals, switching did not interact with the directionality of the switch, in the monolinguals it did (335–395 ms, *p* < 0.03; Fig. 6*F*). This pattern potentially reflects increased activity for the onset of comprehensible speech (i.e., the “turning on” of the language system plausibly elicited the increased activity in the monolinguals).

Additionally, while bilingual speakers showed sensitivity to the type of discourse boundary that housed the language switch, monolinguals did not show such sensitivity (i.e., there was no interaction in the left auditory cortex between boundary type and switching in the monolingual speakers). Given that we did not have sufficient occurrences of switching at each boundary type to enable a further distinction of switches by language, it is not surprising that monolinguals did not show an effect of boundary type: even if they had been able to decode the boundary types in English, they would not have been able to do so in Arabic. Hence, potential sensitivity to boundary type in English may not have been enough to elicit an overall reliable effect of boundary type. Last, and unsurprisingly, we found a main effect of language in the monolingual participants' auditory cortex, which showed increased activity for English compared with Arabic (left auditory cortex: 285–435 ms, *p* < 0.001; right auditory cortex: 235–435 ms, *p* < 0.0001). This contrasts with the results from the bilingual participants, who did not show such activity differences for one language as opposed to the other, presumably due to their being equally engaged by both.

The combination of these results thus suggests that the increased activity observed in the auditory cortices in bilingual speakers indeed reflects speech comprehension after a language switch as opposed to a change in the low-level features of the auditory signal or overall differences between Arabic and English phonemic inventories.

### Representation of interlocutor-type throughout the trial across production and comprehension: MVPA

**Figure 7.**
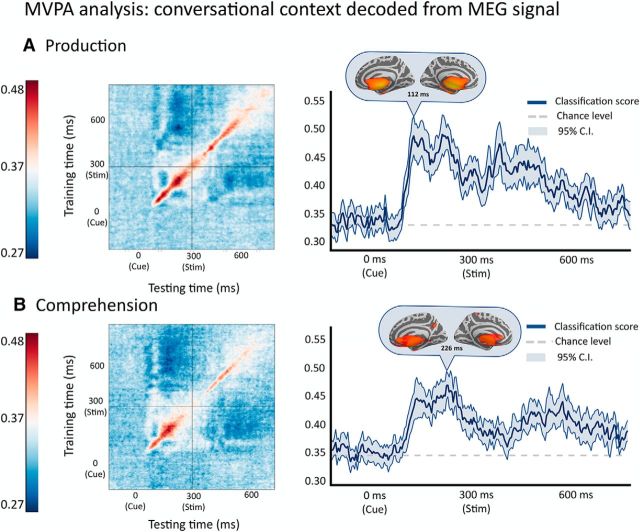
MVPA of context for Production (***A***) and Comprehension (***B***) using Generalization across time (King et al., 2014). ***A***, ***B***, Left panels, Classifier accuracy trained and tested at every time point. Right panels, Classifier accuracy for the diagonal of the matrix (i.e., when the classifier was trained and tested on the same time point). Shading along the decoding accuracy represents 95% CIs. In each panel, a full brain shows the source-localization of the pattern weights at the peak of the classification accuracy.

To further investigate the effect of interlocutor identity, we used an MVPA to test whether interlocutor representations disappear immediately after cue presentation, suggesting that once information about the language these cues represent is extracted, the interlocutor is no longer represented, or whether interlocutor representations persist through the whole trial, suggesting an active role for them in lexical retrieval. A generalization across time procedure (King et al., 2014) (see online Materials and Methods) revealed that the language cue was reliably decoded from the sensor topographies during the whole trial, long after the cue had disappeared from the screen. In production, the classifier performed significantly better than chance starting at 165 ms after presentation of the cue and until 480 ms after the presentation of the picture (for naming), showing that the cue representation remained present even while retrieving the target lexical item (overall significance of classification analysis, *p* = 0.0021 after correction with false discovery rate; Fig. 7*A*). In comprehension, the classifier successfully identified the cue at 170–350 ms after cue presentation (thus extending to 50 ms after the presentation of the auditory word). After this, the accuracy of the classifier lowered to chance at 105 ms after word onset but peaked again starting at 252 ms, suggesting that, although the representation of the interlocutor fades, it is retrieved back upon presentation of the stimulus (overall significance, *p* = 0.0024; Fig. 7*B*). Hence, these results suggest that interlocutor identity is not only used to extract target language information, but rather it is maintained and exploited during lexical retrieval both in production and comprehension.

**Figure 8.**
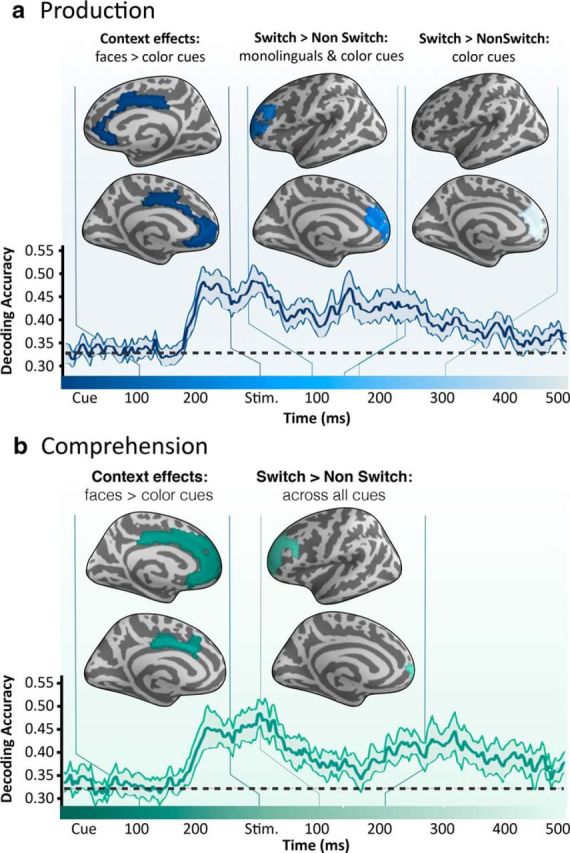
Summary of results for production (***a***) and comprehension (***b***) showing an early effect of Context, time locked to the presentation of the cue, but no effect of switching until the word to be produced or comprehended was revealed. In each panel, a standard free surfer average brain illustrates the extent of each cluster. Vertical lines on each side of the brain indicate the duration of the effect in the horizontal time line. Underneath the average brains, a horizontal line indicates context decoding accuracy at each millisecond: the shading indicates 95% CIs.

In all, as summarized in Figure 8, we observed no evidence of switching until the actual word to be comprehended or produced was revealed, although a representation of the interlocutor type, and consequently target language in the Monolingual and Color-cue contexts, could be decoded from the sensor data starting at 170 ms after cue onset and lasting until 480 ms after word onset, across comprehension and production.

## Discussion

To connect the neurobiology of bilingual language control to its multifaceted, highly adaptative, real-world nature, we sought to characterize the brain basis of language switching in a variety of real or simulated interactional contexts, involving both production and comprehension. In production, as the switching became more natural, the involvement of executive control areas lessened and participants became quicker and more accurate. Further, when language switching was completely spontaneous, the act of switching did not increase activity in prefrontal and anterior cingulate control networks. In comprehension, all the laboratory tasks, with isolated words as stimuli, recruited the dlPFC and ACC, but the understanding of natural switches within a conversation only engaged the auditory cortex. Further, we found that, while bilingual individuals tracked the predictability of the switch, monolingual control participants could not. In all, these results suggest a clear contrast between the perception of natural switches and switches elicited using laboratory paradigms, and provide the first spatiotemporal characterization of how language control networks adapt to different bilingual contexts.

### Language switching in production

When the imagined “conversational” partner of our bilingual participants was another, similarly bilingual individual, our participants produced language switches without any behavioral cost or measurable neural effects in the executive control network. Thus, contrary to the long-held assumption that language switching is effortful (Meuter and Allport, 1999; Costa and Santesteban, 2004), switching may indeed be costless so long as it occurs voluntarily. This finding conforms to bilinguals' intuition that switching is easy with a bilingual conversational partner because this context allows one to choose whatever form is most accessible at any given moment (Kleinman and Gollan, 2016), regardless of its language, resulting in noneffortful retrieval (see also Gollan and Ferreira, 2009, their Experiment 1, their Fig. 2).

In contrast, in the color-cued artificial context, we replicated previous behavioral (Meuter and Allport, 1999; Costa and Santesteban, 2004) and neural (Rodriguez-Fornells et al., 2002; Crinion et al., 2006; Abutalebi et al., 2008; Blanco-Elorrieta and Pylkkänen, 2016) switch effects, with larger signals elicited in the dlPFC and ACC during Switch than NonSwitch trials, followed by longer and less accurate reaction times. Differential engagement for Switch over NonSwitch trials was also identified in the monolingual context, although to a lesser extent, suggesting that the involvement of prefrontal control networks may arise whenever lexical selection is constrained by external factors. However, the fact that the dlPFC and ACC switch effects were longer lasting in the color-cued than in the monolingual context does suggest a difference between the artificial and more natural external cues, where naturality of the cue is defined by its natural relatability to target language. Hence, it would appear that using unnatural cues and paradigms that very closely mirror the type of paradigms used in the general executive control literature (e.g., Monsell, 2003) artificially increases the recruitment of the top-down selection and inhibition networks (MacDonald et al., 2000; Braver et al., 2003; Aron et al., 2004). This supports that ecologically valid paradigms are required to tap into the real mechanisms underlying bilingual language production.

Last, our results constitute the first empirical evidence to support the adaptive control hypothesis proposed by Green and Abutalebi (2013) and show that language control processes adapt to the demands of the interactional context. Specifically, our results suggest that the recruitment of the dlPFC and ACC during language switching may be limited to situations where to accomplish the task goal, conflict needs to be monitored and interference suppressed (Kerns et al., 2004; Abutalebi et al., 2013). These demands emerge when languages are competing (in color-cued and monolinguals contexts) but not when they work in cooperation (bilingual context). Last, as regards the timing of the effects, the neural switch effects in this experiment began earlier than in previous studies (e.g., Blanco-Elorrieta and Pylkkänen, 2016), presumably due to the presentation of the language cue in this study occurring 300 ms before stimulus presentation and in line with studies showing that earlier presentation of the cue leads to faster output (Costa and Santesteban, 2004).

### Language switching in comprehension

Given recent findings that the bilingual brain can proactively use interlocutor identity for predicting an upcoming language before the onset of the auditory-linguistic signal (Martin et al., 2016), our expectation was to observe smaller switch effects for the color-cued and monolingual contexts, which allowed for such prediction, whereas the bilingual context did not. However, the dlPFC and ACC were uniformly recruited across all contexts, supporting that predicting the upcoming language was not sufficient to fully prepare and reduce control demands. One potential reason for this may be the presentation rate of the cue and the picture: It is possible that a longer presentation of the cue would have allowed bilingual individuals to more accurately predict and prepare for the upcoming language.

In contrast, our results showed no increased recruitment of the prefrontal control areas for language switching when the switches were contained within continuous speech. One possibility for the lack of dlPFC and ACC mediation during the comprehension of natural switches is that low-level phonological features may vary immediately before a switch, which participants can register to prepare for the upcoming language switch. Another possibility is that, as bilinguals keep both languages active even when only one is being used (Kroll et al., 2006; for reviews, see Bialystok et al., 2009) and as words in the two languages are stored in an integrated lexicon (BIA+) (Dijkstra and van Heuven, 2002), the governing rules of lexical access during a fully bilingual conversation may have simply been equal to those applied by monolinguals, preventing the need for conflict resolution.

Under the latter type of hypothesis, one could interpret the switch effects in the auditory cortex as *surprisal* effects, where *surprisal* is a particular function of conditional probability of a given word occurring (Hale, 2001). This proposal would align with the assumption that the brain is a predictive system that constantly attempts to improve the predictability of inputs. Under this account, the auditory cortex switch effect would be caused by a reduced prediction for a language switch compared with the prediction of continuing in the same language, which would account for smaller *surprisal* effects at bigger syntactic boundaries in bilinguals, and no sensitivity to boundary type in monolinguals. Effects of *surprisal* have been consistently found in Heschel's gyrus and transverse temporal gyrus (Pallier et al., 2011; Gwilliams and Marantz, 2015; Willems et al., 2015), which accurately overlap with the anatomical location within which our auditory switching effect was observed. This account would additionally predict the lack of *surprisal* effects in single-word presentation because the *surprisal* value is based on the probability of a given word occurring and during single-word presentation, participants could not have had any prediction about upcoming words. Hence, this study raises the possibility that comprehending language switches in a real conversation may be computationally similar to the processing of unpredicted words in a monolingual setting.

Last, we note that the switch effect in the laboratory comprehension tasks in this study extended to the dlPFC, contrasting with Blanco-Elorrieta and Pylkkänen (2016), who found a dissociation between language switching in production and comprehension. In that paradigm, language switching in comprehension was completely bottom-up (i.e., no cue was presented before the auditory stimulus). Hence, it is highly likely that the dlPFC recruitment in the current experiment was due to the presentation of a cue before the stimulus. This is supported by the fact that the dlPFC has been consistently reported to intervene in goal-directed behavior (Hikosaka and Isoda, 2010), and it has been suggested that early activation in response to a cue may be sufficient to trigger its engagement in bilinguals (Luk et al., 2011).

### Processing the identity of the interlocutor

Our results indicate that interlocutor identity was encoded between 100 and 300 ms after the onset of the interlocutor cue, before stimulus presentation. The timing of this effect is remarkably consistent with previous studies that have found sensitivity to interlocutor during this time window, and is also spatially and temporally overlapping with studies that have found the posterior ACC to be sensitive to different cues to language selection (Blanco-Elorrieta and Pylkkänen, 2016; Martin et al., 2016). Hence, the coherence within this set of findings suggests that the posterior ACC plays an important role in interlocutor information processing, converging with literature that relates the posterior ACC to social and emotional processing (Etkin et al., 2011). The combination of these results suggests that the posterior ACC may play a key role in natural bilingual communication by representing and maintaining interlocutor information. This function is paramount given that much of communication relies on bilinguals adequately adapting to the interlocutor with which they are communicating.

Last, the MVPA showed that interlocutor representation can be decoded starting at 160 ms after interlocutor presentation; and more importantly, it revealed that while in production the interlocutor representation is maintained during the whole trial; in comprehension, the representation fades and is later retrieved upon presentation of the auditory stimulus. This suggests that interlocutor reevaluation is relevant for lexical access during auditory comprehension.

In conclusion, this study characterized the changes in the neural regions and circuits associated with bilingual language control processes across different naturalistic interactional scenarios, revealing a dissociation between artificial paradigms and spontaneous bilingual performance. The successful use of a natural conversation to study the comprehension of language switches revealed the auditory cortex as a candidate to house the processing mechanisms of auditory language switches.

## Notes

Supplemental material including 1) Experimental stimulus list, 2) Participants language profile, and 3) Transcription of the natural conversation switches, can be viewed at the website of the Neuroscience of language lab (http://www.psych.nyu.edu/pylkkanen/lab/BlancoElorrieta_Pylkkanen_VoluntarySwitching_materials.html). This material has not been peer reviewed.
